# Model for the Peptide-Free Conformation of Class II MHC Proteins

**DOI:** 10.1371/journal.pone.0002403

**Published:** 2008-06-11

**Authors:** Corrie A. Painter, Anthony Cruz, Gustavo E. López, Lawrence J. Stern, Zarixia Zavala-Ruiz

**Affiliations:** 1 Department of Biochemistry and Molecular Pharmacology, University of Massachusetts Medical School, Worcester, Massachusetts, United States of America; 2 Department of Chemistry, University of Puerto Rico, Mayagüez, Puerto Rico; 3 Department of Pathology, University of Massachusetts Medical School, Worcester, Massachusetts, United States of America; 4 Department of Chemistry, University of Puerto Rico, San Juan, Puerto Rico; University of Arkansas, United States of America

## Abstract

**Background:**

Major histocompatibility complex proteins are believed to undergo significant conformational changes concomitant with peptide binding, but structural characterization of these changes has remained elusive.

**Methodology/Principal Findings:**

Here we use molecular dynamics simulations and experimental probes of protein conformation to investigate the peptide-free state of class II MHC proteins. Upon computational removal of the bound peptide from HLA-DR1-peptide complex, the α50-59 region folded into the P1-P4 region of the peptide binding site, adopting the same conformation as a bound peptide. Strikingly, the structure of the hydrophobic P1 pocket is maintained by engagement of the side chain of Phe α54. In addition, conserved hydrogen bonds observed in crystal structures between the peptide backbone and numerous MHC side chains are maintained between the α51-55 region and the rest of the molecule. The model for the peptide-free conformation was evaluated using conformationally-sensitive antibody and superantigen probes predicted to show no change, moderate change, or dramatic changes in their interaction with peptide-free DR1 and peptide-loaded DR1. The binding observed for these probes is in agreement with the movements predicted by the model.

**Conclusion/Significance:**

This work presents a molecular model for peptide-free class II MHC proteins that can help to interpret the conformational changes known to occur within the protein during peptide binding and release, and can provide insight into possible mechanisms for DM action.

## Introduction

Class II major histocompatibility complex (MHC) are heterodimeric proteins which bind antigenic peptides as part of the adaptive immune response to foreign pathogens. Upon binding peptides derived from endosomes or the extracellular milieu, the intact MHC II-peptide complex is displayed at the cell surface of antigen presenting cells (APC) for surveillance by CD4+ T-cells [1]. Interaction between the APC and its cognate CD4+ T-cell leads to an effector response which then clears the body of the invading pathogen.

Peptides bind to the MHC II in an extended polyproline type II helix along a binding groove contributed to by both the alpha and beta subunits. Crystal studies of allelic variants bound to a variety of peptides has revealed a conserved hydrogen bonding network which exists between the peptide backbone and main chain residues along the helices of the alpha and beta binding domain [2]. Additionally, binding energy is created by the interaction of peptide side chains and pockets within the binding groove of the MHC II binding domain. Residues lining these pockets vary between alleles which thus lead to tremendous diversity within the peptide repertoire. Generally, these pockets accommodate residue side chains from the peptide at the P1, P4, P6 and P9 positions with smaller pockets or shelves in the binding site accommodating the P3 and P7 residues; these pockets are numbered along the peptide relative to a large usually hydrophobic pocket near the peptide binding site. For DR1 (DRB1*0101), a common human class II MHC protein and the subject of this study, the P1 pocket shows a strong preference for large hydrophobic side chains (Trp, Tyr, Phe, Leu and Ile), the P6 pocket has a strong preference for smaller residues (Gly, Ala, Ser and Pro) and the P4 and P9 pockets have weaker preference for residues with some aliphatic character [3].

Although there is little structural variation observed among crystal structures determined for MHC II-peptide complexes, numerous studies have reported alternate conformations for particular MHC II-peptide complexes [4], [5], [6], [7] and for peptide-free MHC II molecules [8], [9]. Peptide-free DR1 has been shown to have a larger hydrodynamic radius than the peptide loaded form (29 vs 35 Å), as well as a decrease in helicity as measured by circular dichroism [9], [10]. These differences are reversed upon binding peptide. Peptide binding and dissociation experiments have shown that peptide-free MHC II can adopt two interconverting forms, one receptive to and one averse to peptide loading [11], [12], [13]. The receptive state is able to bind peptide rapidly, but will convert to the peptide averse form within minutes if not stabilized by peptide or by association with the chaperone HLA-DM. It has been proposed that HLA-DM mediates its function by shifting the equilibrium of peptide averse to a peptide receptive state; however, the peptide loading process is still relatively undefined [14], [15]. In order to gain a better understanding of this process, it is necessary to develop a more detailed understanding into the structural changes that exist based on peptide occupancy.

Previous work using conformationally sensitive monoclonal antibodies raised against the β chain of DR1 has revealed that a noncontiguous epitope in the peptide binding region (residues β53–67) and another in the lower Ig-like domain (residues β186–189) are accessible only in the peptide-free conformation [16]. Another study which used differential chemical modification found residues α50,67, β98,189 selectively modified in peptide-free but not peptide-loaded DR1 [17]. Although these studies helped to define regions within the structure that change upon the peptide occupancy state, there is not enough information to generate a working model of the peptide-free DR1. Molecular dynamics simulations have been used to gain insight into conformational changes relative to an experimentally defined structure [18], [19]. Combined with experimental support, models developed from this method can be substantiated. In this study, we performed molecular dynamics simulation of DR1 in both the peptide-free and peptide-loaded states. Several regions of DR1 were predicted by this analysis to change conformation substantially upon loss of bound peptide. Differential binding of conformationally-specific antibody and superantigen probes to the peptide-free and peptide-loaded forms of DR1 provides experimental support for this model.

## Methods

### Generation of DR1

DR1 was expressed, purified and folded as previously described [20]. Briefly, the extracellular domains of DR1 subunits were expressed individually as inclusion bodies in *Escherichia coli*. Subunits were purified by anion exchange chromatography and then diluted into folding buffer in the presence or absence of a 5 fold molar excess of the HA peptide (PKYVKQNTLKLAT). Folded peptide-free or peptide-loaded DR1, was purified by affinity chromatography on an LB3.1-protein column and by gel-filtration chromatography to remove any aggregated protein. Pooled fractions were concentrated to 1 mg/ml and stored at 4°C. For some SPR studies, DR1 with a cysteine modification at the C-terminus of the alpha subunit was prepared by expression in insect cells as previously described [21] Briefly, DR1-α_cys_ was expressed in S2 insect cells in serum free medium and purified from culture supernatant by LB3.1 affinity and gel filtration chromatography. This material is substantially free of peptide [22]. To generate loaded complex, purified DR1α_cys_ was incubated with excess peptide in binding buffer (100 mM phosphate pH 5.0, 0.02% NaN_3_, 1 mM EDTA, 50 mMNaCl, 0.05% octyl glucoside, 2 mM dithriothreitol (DTT), 0.01 mg/ml PMSF) for 3 days at 37°C. Peptide-free and peptide-loaded DR1α_cys_ were purified as described above for *E. coli* derived protein.

### ELISA

A sandwich ELISA was used to measure binding of peptide-free or peptide-loaded DR1 to LB3.1 and MEM264 as previously described [22]. Plates were developed with ABTS and read on a Polarstar plate reader (BMG Labtech) at 405 nm absorbance. Half-maximal binding concentrations were obtained from a 4-parameter binding equation fit to the data.

### Biacore

We used a BIAcore 2000 surface plasmon resonance biosensor for the SEC3 experiments and a Biacore 3000 for all other SPR experiments (Biacore AB, Uppsala, Sweden). Biotinylated DR1 was immobilized using Sensor Chip SA. All other experiments used standard carbodiimide-mediated amine coupling to Sensor Chip CM-5. Data were background-subtracted using an unmodified reference surface. The data were fit to the 1∶1 binding with baseline drift model provided by BiaEval software. Fit residuals are shown in the figure and generally represent <10% of the overall signal. The LB3.1 and MEM264 were regenerated with 20 mM NaOH for 1.5 or 1 minutes respectively, followed by a 2 minute stabilization in running buffer. For the other SEC3 and DR1 surfaces, bound protein eluted rapidly and a separate regeneration protocol was not needed.

### Molecular Dynamic Simulation

Each simulated system consisted of one protein molecule in a cubic box with a distance of 3.0 nm from its periodic image, with approximately 30,200 molecules of water. Coordinates for the peptide-bound form of DR1 were downloaded from the Protein Data Bank (PDB code: 1SJE) [23]. The peptide-free form of the DR1 was prepared by removing the sixteen residue peptide from the peptide-binding grove. SEC3 was removed from both structures.

The energy of the system was minimized with the steepest descent algorithm, followed by a 600 ps of positional restraint dynamics to generate the starting point for molecular dynamics. The GROMOS9643a1 force field [24] was used, and all simulations were carried out at constant temperature (298 K), pressure (1 atm), and number of molecules using a Berendsen weak coupling bath with a coupling constant of 0.1 ps for temperature and 0.5 ps for the pressure [25]. A twin range cut-off of 0.9/1.4 nm for van der Waals interactions was applied, and the particle mesh Ewald algorithm was used for long range electrostatic interactions [26]. Neighbor lists were utilized and updated every five steps, and all protein and water bond lengths were constrained using the LINCS and SETTLE algorithm, respectively [27].

The length of the simulation was determined by monitoring the convergence of various mechanical properties of the system. The simulation was stopped when the value for the root mean square deviation (RMSD) did not fluctuate more than 3.0 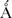 from its average value during 2 ns. As described [28], if the simulation reached an RMSD that oscillates around a constant value, it can be assumed the system has converged to a stable or a metastable structure. For both systems, the simulations were terminated after 60 ns with a time step of 2 fs. The coordinates were saved every five picoseconds and the analysis was performed using GROMACS v.3.2.1 simulation package [29]. All molecular graphics images were generated using the Visual Molecular Dynamics (VMD) software [30].

## Results

### Modeling the structure of the peptide-free form of HLA-DR1 by molecular dynamics

**Figure 1:**
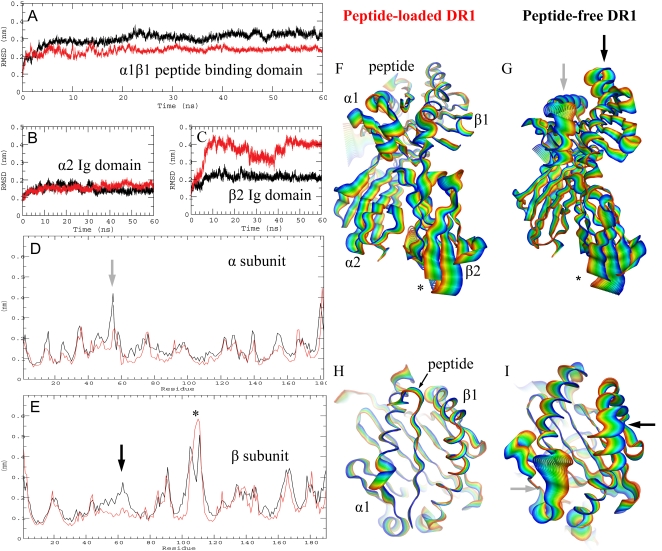
Molecular dynamics simulation of peptide-bound and peptide-free DR1. A, (A–C) RMS deviation over time for the peptide-loaded (red) and peptide-free (black) simulations, for the α1β1 peptide-binding domain (A), the Ig-like α2 domain (B), and the Ig-like β2 domain (C). D–E, Root mean square (RMS) fluctuation during the simulation for each residue (all atoms included) for the α (D) and β (E) subunits. (F–I) Molecular dynamic trajectories for the peptide-loaded (F,H) and peptide-free (G,I) form of DR1. Initial states shown in blue, final states shown in red, with a linear interpolation of conformations between the initial and final structures shown in other colors. (F,G), Side view of entire protein, (H,I) Top view of peptide binding site only.

A model for the peptide-free structure of DR1 was obtained by molecular dynamics simulation starting from the X-ray coordinates of a DR1-peptide complex (PDB Code: 1SJE, [31]) from which peptide was removed before the start of the simulation. A parallel simulation was started from the same coordinates but without the removal of peptide. Simulations were carried out in a water-filled box at constant temperature and pressure using the GROMOS force field [29] and 2 fs time steps. During the simulation, the total root mean square deviation, RMSD, from the starting coordinates was followed in various regions of the protein, Fig. 1A–1C. Large changes in RMSD were observed over the first 5–10 ns, particularly in the α1β1 peptide binding domain and the lower β2 immunoglobulin-like domain, with much smaller fluctuations occurring thereafter. The models were investigated in detail at the 10 ns time point, as described below.

RMS fluctuations during the first 10ns of the peptide-loaded and peptide-free simulations are shown in Fig. 1D and E as a function of residue number. A trajectory of the simulation is depicted in Fig. 1F for the peptide-free and in Fig. 1G for the peptide-loaded dynamics runs. Panels H and I show a different view highlighting the peptide binding region. In the peptide-binding site, significant movements can be seen in the α-helices of the peptide-free form, as compared to those of the peptide-loaded form, which do not fluctuate or move as much. In the α subunit, the principle differences between the peptide-loaded and peptide-free simulations were in the region α34-60 (light arrows). This region, part of the peptide binding domain, corresponds to the last two strands of the beta sheet “floor” and the first half of the α helical region forming one side of the peptide binding site. In the β subunit the largest RMS fluctuations were in the lower β2 immuoglobulin domain, residues β90–110, and were observed in both peptide-loaded and peptide-free simulations (Fig. 1C and E, asterisk). Different orientations of the β2 domain relative to the peptide binding domain already have been observed in different HLA-DR1 crystal structures, reflecting orientational flexibility in this domain (Fig. S1). Elsewhere in the β-subunit, large RMS fluctuations were observed for the region β50-70 in the peptide binding site. In this region larger deviations were observed in the peptide-free as compared to the peptide-loaded simulation (Fig. 1F–I). A difference distance matrix plot [32] calculated at 10ns highlights the regions that are different in this simulated peptide-loaded and peptide-free forms (Fig. S2).

### Motion of the α50-59 region into the amino-terminal end of the peptide binding site

**Figure 2:**
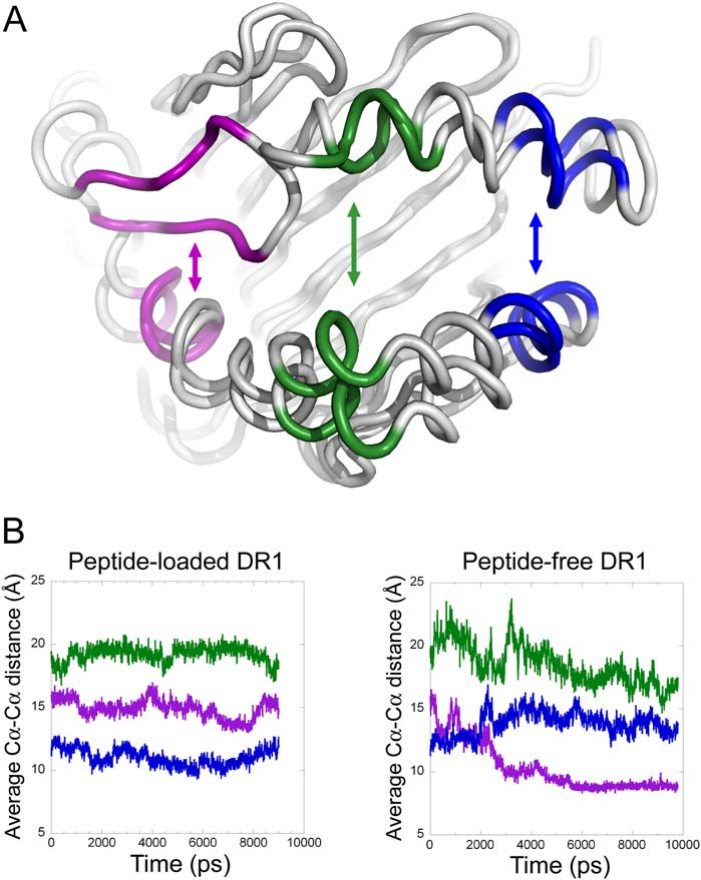
Motion of a50-59 into the peptide binding site during molecular dynamics in the absence of peptide. (A), Ribbon diagram of peptide-free DR1 before and after molecular dynamics simulations, showing residues at N-terminal end (magenta, α52-57 and β79-83), central region (green, α62-66 and β65-69), or C-terminal end (blue, α71-75 and β58-62) of peptide binding site, used to calculate distances across the binding site (indicated by arrows). (B), Changes during molecular dynamics simulation of distances across the peptide binding site at three different locations, colored as indicated in panel A.

**Figure 3:**
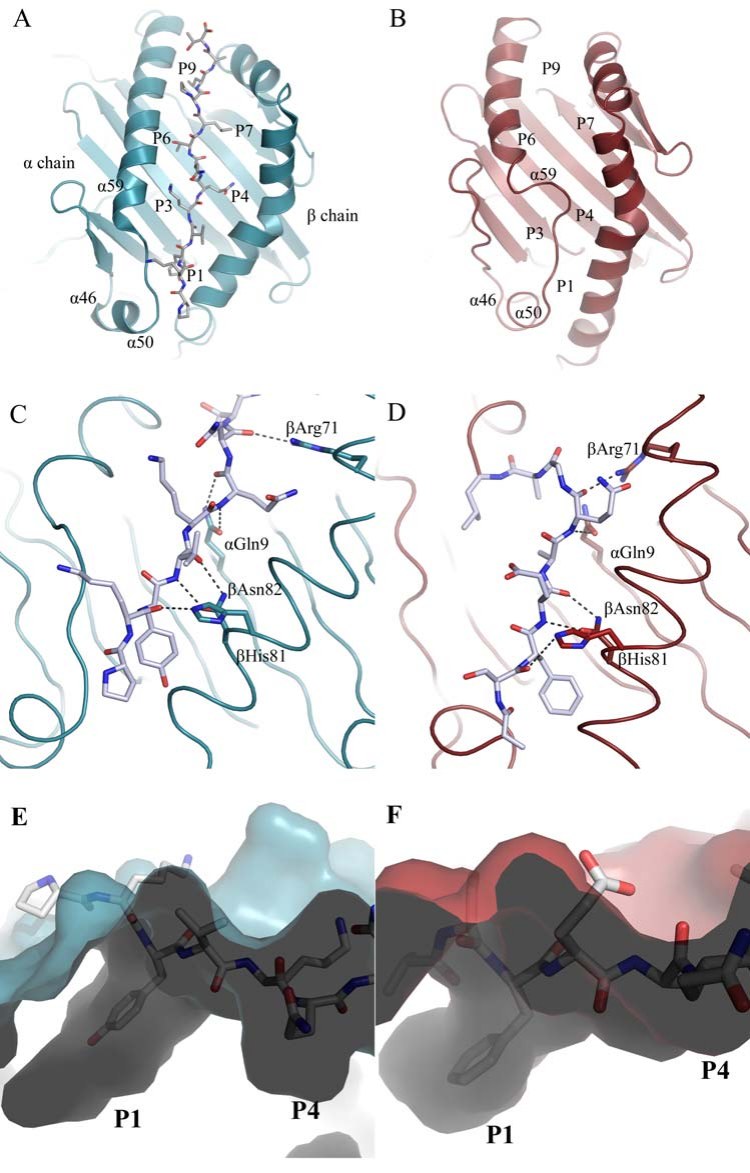
Comparison of models for peptide-loaded and peptide-free DR1. A, Ribbon diagram of DR1 (blue) bound to the HA-peptide. The peptide is shown as stick with carbon atoms in white; and nitrogen and oxygen atoms in blue and red, respectively. Pocket locations within the peptide-binding site are labeled. B, Ribbon diagram of the peptide-free HLA-DR1 (red). C,D, hydrogen-bonding interactions for the peptide-loaded (C) and peptide-free (D) proteins. E,F Surface side-view of the N-terminal region of the peptide-binding site for the peptide-loaded (E) and peptide-free (F) proteins. In (D and F), α50-59 of the peptide-free form is shown in stick representation as for the HA peptide. Figures were generated using PyMol [58]

The major conformational alteration observed during the simulations is a narrowing of the N-terminal region of the peptide-binding site (Fig. 2A). This narrowing of the site occurred early during the dynamics simulations run in the absence of peptide, and persisted throughout the entire 60ns time course. Such narrowing was not observed during dynamics simulations of the peptide-loaded form (Fig. 2B), although at late time points (>15 ns) the peptide-loaded simulation occasionally sampled conformations having some characteristics of the peptide-free form (not shown). In the model of the peptide-free form of DR1 derived by molecular dynamics simulation, the peptide binding site is dramatically altered as compared to the highly stereotyped conformation observed in crystal structures of the peptide-bound form. During the simulation, the α50-59 region of DR1 moves to fill the amino-terminal end of the peptide-binding site occupying, in part, the area where the antigenic peptide is usually found (Fig. 3). The amino-terminal region of the peptide-binding site of HLA-DR1 was previously suggested to be more flexible upon peptide-removal in a normal mode analysis [33] as well as in another molecular dynamics calculation [34]. In our model, a sharp kink forms at Gly α58, allowing the region α50-59 to fold into the binding site, taking the place of the bound peptide in the P1 to P4 region. Smaller changes but still significant changes are observed in the helical regions that flank α50-59, in the adjacent α46-49 loop, and in beta subunit helical regions (compare Fig. 3A and B).

After moving into the peptide-binding site, the main chain of the α50-59 region is able to satisfy essentially all of the hydrogen bonds in this region lost upon removal of the peptide. In the conserved arrangement observed in class II MHC-peptide crystal structures, the backbone of a bound antigenic peptide forms six hydrogen bonds with the side chains of non-polymorphic DR1 residues Gln α9, Arg β71, His β81 and Asn β82 (Fig. 3C). In the molecular dynamics model of the peptide-free form of DR1, each of these hydrogen-bonding interactions is observed, by direct hydrogen bonding between the main chain atoms of DR1 α53-57 and side chains of Gln α9, Arg β71, His β81 and Asn β82 (Fig. 3D).

Movement of the α50-59 region into the peptide binding site also results in occupancy of the P1-P4 side-chain binding pockets. These pockets line the peptide binding site, and accommodate side chains of the bound peptide. The P1 pocket of DR1 is the major determinant of peptide binding, and usually accommodates a large hydrophobic side chain [10]. In the molecular dynamics model of the peptide-free form of DR1, the side chain of Phe α54 binds into the P1 pocket, in the same orientation as observed for an aromatic P1 residue from a crystal structure of peptide-loaded DR1 complexes (Fig. 3E and F). The P4 pocket is shallower than the P1 pocket and open at the end, and in DR1 exhibits a weaker preference for residues with some aliphatic character. In the molecular dynamics model of the peptide-free form of DR1, the side chain of Gln α57 binds into the P4 pocket, essentially identical to Gln at the corresponding position in a bound peptide.

### Experimental probes of conformational differences between peptide-free and peptide-loaded HLA-DR1

We attempted to evaluate whether or not the striking alterations predicted by the molecular dynamics simulations corresponded to actual conformational differences between peptide-free and peptide loaded proteins, using conformation-specfic superantigen and antibody probes of DR1 structure. Superantigens are soluble bacterial toxins that bind to class II MHC proteins and T cell receptors, causing polyclonal T cell activation independent of peptide antigen [35]. Superantigen binding sites on the DR alpha subunit have been determined by X-ray crystallography of superantigen-MHC-peptide complexes [36], but superantigen binding to peptide-free DR1 has not been described. Conformation-specific antibody probe of DR1 structure also are available, although the binding sites have been characterized only by domain-swap or mutagenesis experiments. In general, most of these antibodies bind to both peptide-free and peptide-loaded DR1 [22]. MEM-264 and the related MEM-265, MEM-266, and MEM-267 antibodies [16] are exceptions to this pattern, in they preferentially bind to peptide-free DR1 Although differential binding to peptide-free and peptide-loaded DR1 has been described for these antibodies, there has not been a quantitative comparison. Finally, LB3.1, a commonly used anti-DR antibody, also has been reported to exhibit differences in reactivity with peptide-free and peptide-loaded DR1 [16], although again a quantitative analysis has not been reported. To evaluate in a quantitivate manner the differential binding of these conformationally-specific superantigen and antibody probes, we used surface plasmon resonance (SPR) to characterize their binding to to peptide-free and peptide-loaded DR1 prepared by refolding purified DR1 alpha and beta subunits in the absence or presence of peptide (see Methods). As previously observed [9], the peptide-free and peptide-loaded preparations exhibited characteristic differences in hydrodynamic radius and stability to SDS-induced subunit dissociation [20] (Fig. S3).

#### Staphylococcal Enterotoxin C3 (SEC3)

**Figure 4:**
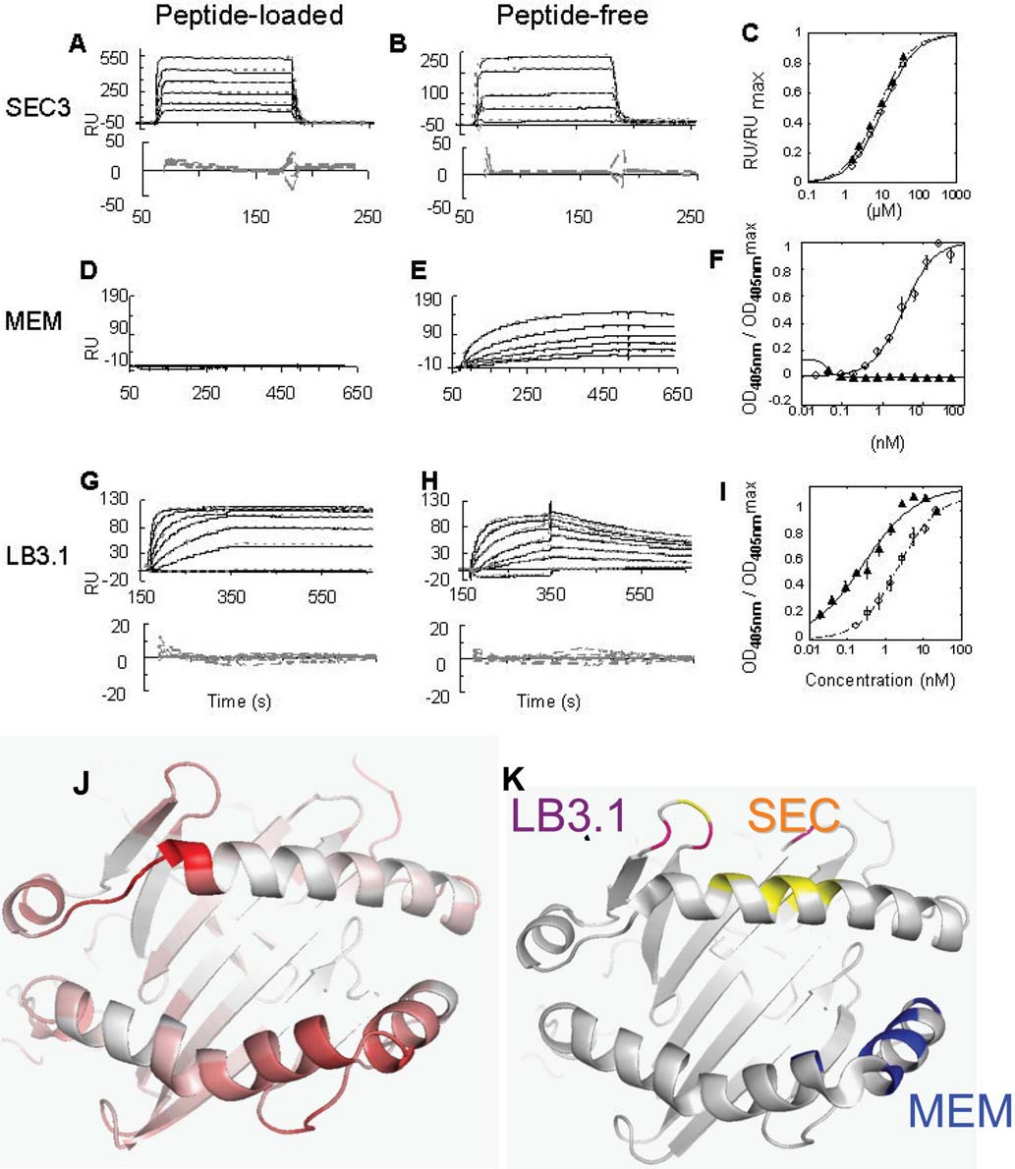
Binding of peptide-loaded and peptide-free DR1 to conformationally sensitive probes. (A–C) SEC3 binding to immobilized bio_DR. A) SPR using peptide-loaded DR1. B) SPR using peptide-free DR1. Residuals shown below SPR traces C) Equilibrium RU values vs. DR concentration, open circles, peptide-free DR1, closed triangles, peptide-loaded DR1. (D–F) DR1 binding to immobilized MEM264. D) SPR using peptide-loaded DR1. E) SPR using peptide-free DR1. F) ELISA of MEM264 binding empty, open circles, and loaded, closed triangles, DR1. (G–I) DR1 binding to immobilized LB3.1. G) SPR using peptide-loaded DR1. (H) SPR using peptide-free DR1.Residuals shown below. (I) ELISA of immobilized LB3.1 binding to peptide-free (open circles) and peptide-loaded (closed triangles) DR1. (J–K) Comparison of experimental to model data. J, Ribbon diagram of the peptide-binding site of DR1, colored by peptide-free to peptide-loaded Cα distances at the end of the dynamics runs. Larger distances shown in darker red. K, same view but colored to indicate sites of conformational probe interaction. The yellow region represents DR1 residues that interact with the superantigen SEC3, magenta resides that interact with the conformationally sensitive antibody LB3.1, and blue residues in the peptide-free protein that interact with the antibody MEM264 specific for the peptide-free protein.

The interaction of the bacterial superantigen SEC3, a tight-binding derivative of staphylococcal enterotoxin C3 (SEC3), with peptide loaded DR1 has been characterized previously by X-ray crystallography and SPR experiments [37]. Contact residues between SEC3 and peptide-loaded DR1 have been mapped by crystallography and by mutagenesis, and include Tyr13, Asp17, Gln18, Met36, Ala37, Leu60, Ile63, Ala64, and Lys67 on the α chain [36]. None of these residues move appreciably during molecular dynamics simulation of the empty protein. To evaluate this experimentally, peptide-free and peptide-loaded DR1 were immobilized to a streptavidin-dextran surface using a C-terminal biotin [21], and binding to varying concentrations of soluble SEC3 was followed by SPR. Essentially identical SEC3 binding behavior was observed for peptide-free and peptide-loaded DR1 (Figure 4A–C). SPR data were fit to a kinetic model, yielding apparent K_D_ values for peptide-free and peptide-loaded DR1 of 14 µM and 11 µM, respectively. Equilibrium analysis of these same data yielded apparent K_D_ values of 6.1 and 8 µM. These values are similar to a previously reported K_D_ value for peptide-loaded DR1 binding to SEC3, 4.6 µM [37]. To confirm these results, we performed this experiment in the opposite orientation, with SEC3 immobilized via standard amine coupling and with peptide-free or peptide-loaded DR1 in the mobile phase. Again, we obtained similar K_D_ values for peptide loaded and peptide-free DR1 6.0 and 6.1 µM, respectively (Table 1). Overall these data demonstrate that SEC3 binding does not distinguish between peptide-loaded and peptide-free DR1. Because this epitope is not predicted to move upon release of peptide, these observations are consistent with the model.

**Table 1:** Antibody and Superantigen Binding Properties of Peptide-loaded and Peptide-free HLA-DR1.

Assay	Parameter	Peptide-loaded DR1^1^	Peptide-free DR1^1^
SEC3-DR1 interaction			
SPR^2^ (n = 1)	k_on_ (M^−1^ s^−1^)	22,000 (1270)	18,000 (1900)
	k_off_ (s^−1^)	0.25 (0.01)	0.25 (0.01)
	K_D_ 3 (nM)	11,000 (1140)	15,000 (1150)
	K_D_ 4 (nM)	6100 (500)	8000 (200)
SPR^5^ (n = 1)	k_on_ (M^−1^ s^−1^)	1930 (88)	1900 (19)
	k_off_ (s^−1^)	0.012 (0.001)	0.012 (0.001)
	K_D_	6000 (300)	6100 (110)
MEM264-DR1 interaction			
SPR^6^ (n = 2)	k_on_ (M^−1^ s^−1^)	Not observed	10,500 (5800)
	k_off_ (s^−1^)	Not observed	9000
	K_D_ (nM)	>2000	24 (11)
ELISA^6^ (n = 3)	C_1/2_ 7 (nM)	>2000	5.3 (0.7)
[16]	C_1/2_ 7 (nM)	Not determined	31
LB3.1-DR1 interaction			
SPR^8^ (n = 4)	k_on_ (M^−1^ s^−1^)	554,000 (10000)	45,600 (8200)
	k_off_ (s^−1^)	0.00017 (0.00006)	0.00165 (0.00017)
	K_D_ (nM)	2.3 (0.5)	76 (6.6)
ELISA^8^ (n = 3)	C_1/2_ 7 (nM)	0.47 (0.15)	2.1 (0.39)

1 Values in parentheses indicate standard errors.

2 Immobilized biotinylated-DR1 with SEC3 in the mobile phase.

3 K_D_ determined by kinetic analysis of on and off rates.

4 K_D_ determined from Scatchard analysis of the equilibrium values.

5 Immobilized SEC3 with DR1 in the mobile phase.

6 Immobilized MEM264 with DR1 in the mobile phase.

7 Concentration required for half-maximal binding signal.

8 Immobilized LB3.1 with DR1 in the mobile phase.

#### Monoclonal antibody MEM-264

MEM-264 is a monoclonal antibody that previously has been shown to bind specifically to the peptide-free conformation of DR1 [16]. The MEM-264 epitope has been mapped by overlapping peptides and alanine scanning mutagenesis, and corresponds to a discontinuous region on the β subunit helical region including residues 53–67 [16]. In the molecular dynamics model for peptide-free DR1, this entire region is predicted to move significantly relative to the rest of the β subunit. We performed a SPR binding analysis with immobilized MEM-264 and either peptide-free or loaded DR1 in the mobile phase (Fig. 4D and E). We found robust binding to immobilized MEM-264 for peptide-free DR1, with an apparent K_D_ of approximately 24 nM. Peptide-loaded DR1 did not bind to MEM-264 at concentrations up to 2 µM. In addition, we compared binding of peptide-free and peptide-loaded DR1 using an equilibrium sandwich ELISA assay (Fig. 4E). Half maximal binding of peptide-free DR1 to MEM-264 was observed at 5.3 nM, whereas no binding was observed for peptide-loaded DR1 at concentrations up to 2 µM. Overall these data suggest that peptide-loaded DR1 binds to MEM-264 at least 400-fold more weakly than does peptide-free DR1.

#### Monoclonal antibody LB3.1

LB3.1 is a conformationally sensitive monoclonal antibody which has three residues known to be important in the binding interaction, all in the loop region proximal to the α50-59 strand [38]. During the simulation, Gln α18 does not move appreciably, Met α36 moves ∼5 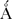 but similarly in the peptide-loaded and peptide-free models, and Lys α39 moves and packs differently against the alpha-subunit helix in the peptide-free and peptide-loaded models. Of these, Gln α18 and Met α36 also are in the SEC3 epitope. Differential binding of LB3.1 to peptide-free and peptide-loaded DR1 has been observed previously [16] but not quantified. We used SPR and ELISA to quantify differences in LB3.1 binding to peptide-free and peptide-loaded DR1. SPR analysis using immobilized LB3.1 and serial dilutions of peptide-loaded and peptide-free DR1 indicated a significant difference in binding kinetics (Fig 4G and H), with tighter binding of the peptide-loaded form. Kinetic analysis revealed apparent K_D_ values of 76nM for peptide-loaded DR1 and 2.3nM for peptide-free DR1. We used ELISA to evaluate the binding interaction under equilibrium conditions (Fig. 4I). Once again, tighter binding of LB3.1 to the peptide-loaded form was observed, with half-maximal binding observed at 0.47 nM and 2.1 nM for the peptide-loaded and peptide-free species respectively (Table1). Overall, these experiments confirm that peptide-free and peptide-loaded DR1 have distinct binding affinities for LB3.1.

## Discussion

Previous work has shown that peptide-free and peptide-loaded forms of DR1 have different physical characteristics, including hydrodynamic radius, chemical and thermal stability, and far-UV CD and near-UV fluorescence spectra [9]. In general, these changes can be induced by binding a wide variety of peptides regardless of length, affinity, or sequence characteristics, provided that the P1 site is occupied [10], [39]. These observations support the notion that there are conformationally distinct forms of the protein based on peptide occupancy [9], [11], [40], [41], [42], [43], [44]. Our goal in this work was to gain insight into structural changes that can occur in the absence of bound peptide. Because conditions for crystallization of peptide-free DR1 have yet to be determined, it is necessary to approach structural questions by alternative methods. Using molecular dynamics in conjunction with experimental data can help to elucidate particular structural events that might otherwise remain obscure.

Two important features of the interaction of peptides with class II MHC proteins have been identified: hydrogen bonds between conserved MHC residues and the peptide main chain amides, and binding of several peptide side chains into pockets in the MHC peptide binding site [2]. Hydrogen bonding interactions involving the peptide N-terminal region in general appear to be more important than those involving the C-terminal region [2], and for DR1 variants the P1 pocket dominates the overall peptide binding behavior [10], [45], [46]. In the molecular dynamics model for the peptide-free form of DR1, the key hydrophobic P1 pocket becomes engaged by Phe α54, and the secondary pocket P4 by Gln α57. Conserved MHC hydrogen bonding residues along the peptide binding groove become engaged by the α50-59 loop region. By the end of the simulation, the peptide-free DR1 has satisfied all of the N-terminal binding interactions that were lost by initial removal of the peptide in a manner consistent with the original bonds made by the peptide-DR1 interaction.

We used conformationally-dependent antibody and superantigen probes in order evaluate the concordance of the model with experimental data. Overall, there is good agreement between the predicted MHC backbone movement and the degree to which the probes are sensitive to the presence or absence of peptide (Fig. 4J and K). The SEC3 epitope is predicted to remain stationary, and our data show no significant differences in binding to peptide-loaded and peptide-free DR1. The MEM264 epitope overlaps with an area in the peptide binding region predicted to undergo a large global movement upon peptide release, and previous results [16] and those shown here demonstrate a dramatic difference in the reactivity of this antibody for peptide-loaded and peptide-free DR1. The LB3.1 epitope has not been comprehensively mapped, although a few residues within the epitope have been identified using DR-IE (human-mouse) chimeric molecules [38] Residues Gln α18 and Met α36 are also included in the SEC3 epitope, but Lys α39 is unique to the LB3.1 epitope. This residue undergoes a rigid body as well as a side chain reorientation movement in the model. Partial shielding of the residue could account for the observed difference in LB3.1 binding to peptide-free and peptide-loaded DR1, although it is possible that other residues not yet identified contribute to LB3.1 binding. Overall, the observed binding to peptide-free and peptide-loaded DR1 for each of the conformational probes that we tested was consistent with predictions from the molecular dynamics models for the peptide-free protein. While this concordance indicates that molecular dynamics may provide a useful model for the peptide-free conformation, none of the available conformational probes directly addresses the key prediction of motion of α50–59 into the peptide binding site, which remains to be validated experimentally.

Peptide-free DR1 has been shown to exist in at least two kinetically defined states, peptide receptive and peptide averse [9], [10], [11], [13], [40]. The peptide receptive form is observed immediately after release of a bound peptide, and is characterized by rapid binding of added peptide. In the absence of peptide, the peptide-receptive form converts to the peptide-averse form, with t_1/2_∼min [11]. The peptide-averse form binds peptide much more slowly, in a process that requires a slow unimolecular conformational change [13]. The predicted conformational change in the peptide-free model could account for the peptide averse form of DR1. It is plausible that upon peptide release, the binding groove is not obstructed and therefore could bind subsequent peptide directly, whereas once the peptide binding groove is engaged by the α50-59 region, the protein would be in the peptide-averse form, and would have to undergo a conformational change in order to allow space for the peptide to enter the binding groove.

In addition to catalyzing peptide binding and release, the peptide exchange factor HLA-DM has also been shown to stabilize the peptide-receptive form of MHC II and to prevent conversion to the inactive form [14], [15], [47], [48]. A key residue in the interaction with DM and DR1 is DR1 Phe α51. When Phe α51 is mutated to Val or Ser the catalytic ability of DM is abolished [49]. Interpreting this result in light of the dynamics model, interaction of DM with this residue could prevent the α50-59 region from engaging the peptide binding groove, inhibiting formation of the averse form. Interestingly, occlusion of the P1 pocket of DR1 by Glyβ86Tyr also has been reported to abolish DM activity [39], [50]. Such a mutation might prevent α50-59 closing into the peptide binding site, and in fact reduced formation of a peptide-averse state has been reported for this mutant [39], [50].

The structures of class I and class II MHC proteins are similar, despite having different domain/subunit organizations and different modes of peptide binding [51]. The α50-59 region, proposed herein to occupy the HLA-DR peptide binding site in the absence of peptide, represents a prominent difference between the structures of class I and class II MHC proteins [51]. In class I MHC proteins, the α1 helix continues unbroken through this region, extending from residue 56 (equivalent to HLA-DR α52) to residue 86 (equivalent to HLA-DR α78), whereas in class II MHC proteins, this region is an extended strand interrupting flanking helical regions. This structural difference may be related to differences in the conformational changes induced in the absence of peptide, which have been characterized for both class I [52], [53] and class II MHC proteins [9], [42], [43]. A crystal structure of a class I MHC protein with a pentapeptide epitope occupying only the C-terminal end of the binding site [54] and molecular dynamics studies of peptide-free class I MHC proteins [55], [56] suggest that for class I MHC proteins the N-terminal side of the peptide binding site could be conformationally stable in the absence of peptide, with conformational changes predicted in the C-terminal end [55], [56]. By contrast, crystal structures of class II MHC-peptide complexes carrying truncated penta-, hexa-, and hepta-peptide analogs occupy the N-terminal side of the peptide binding site, leaving the C-terminal end empty but not conformationally altered [57]. Recently, a study of dipeptide-triggered ligand exchange of HLA-DR1 suggested that short peptides can prevent closure of the N-terminal side of the peptide-binding site predicted by molecular dynamics to occur in the absence of peptide [34]. Finally, normal mode analysis of HLA-A2 [33] and HLA-DR1 [33] also highlighted differences between their potential dynamic motions, with the N-terminal side of the binding site predicted to be more flexible for class II MHC proteins and the C-terminal side more flexible for class I MHC protein.

In summary, this work presents a molecular model for the conformational change induced by peptide removal from an MHC II protein. The model was derived from molecular dynamics calculations and tested using experimental conformational probes. Development of a model for the peptide-free form of DR1 can help to interpret the conformational changes known to occur within the protein during peptide binding and release, and can provide insight into possible mechanisms for DM action.

## Supporting Information

Figure S1 Conformational variation in DR1 crystal structures. A, Overlay of crystallographically distinct αβ heterodimers from three crystal structures reported for DR1-peptide complexes solved in the absence of superantigen. B, RMS Cα distances for aligned αβ heterodimers, with mean RMS in each domain indicated. Structures aligned using LSQMAN [59].
(4.36 MB TIF)

Figure S2 Difference distance matrix between the peptide-loaded and peptide-free conformations of HLA-DR1. The blue squares show areas in the protein that move away from each other in the peptide-free model when compared to the peptide-loaded conformation, with stronger blue intensity for the areas that are the further apart (15 Å difference) and lighter blue for the areas that move away but not as much (4 Å difference). The red squares show regions on the peptide-free form that move closer to other regions in the protein when compared to peptide-loaded HLA-DR1; intense red color are areas that move more. The gray squares are regions that do not change as much (0–3.99 Å).
(4.79 MB TIF)

Figure S3 Characterization of peptide loaded and peptide-free DR1. A, analysis of peptide-free DR1 and peptide-loaded DR1 by gel filtration (Superdex 200). Peptide-free DR1 (dotted line) has a larger hydrodynamic radius the peptide-loaded DR1(solid line). Arrows indicate position and molecular weight of standard proteins. X axis represents time in minutes, Y axis represents optical density (milli OD). B, 12% SDS-PAGE analysis of peptide-free DR1 and peptide-loaded DR1. Peptide-free DR1 dissociates into alpha beta subunits in SDS whereas peptide-loaded DR1 is resistant to SDS dissociation until boiled.
(3.27 MB TIF)
