# Supplementary figures and images for: Model for the Peptide-Free Conformation of Class II MHC Proteins
Source: PLoS One. 2008 Jun 11;3(6):e2403. doi: 10.1371/journal.pone.0002403 (PMC2408972; doi:10.1371/journal.pone.0002403)

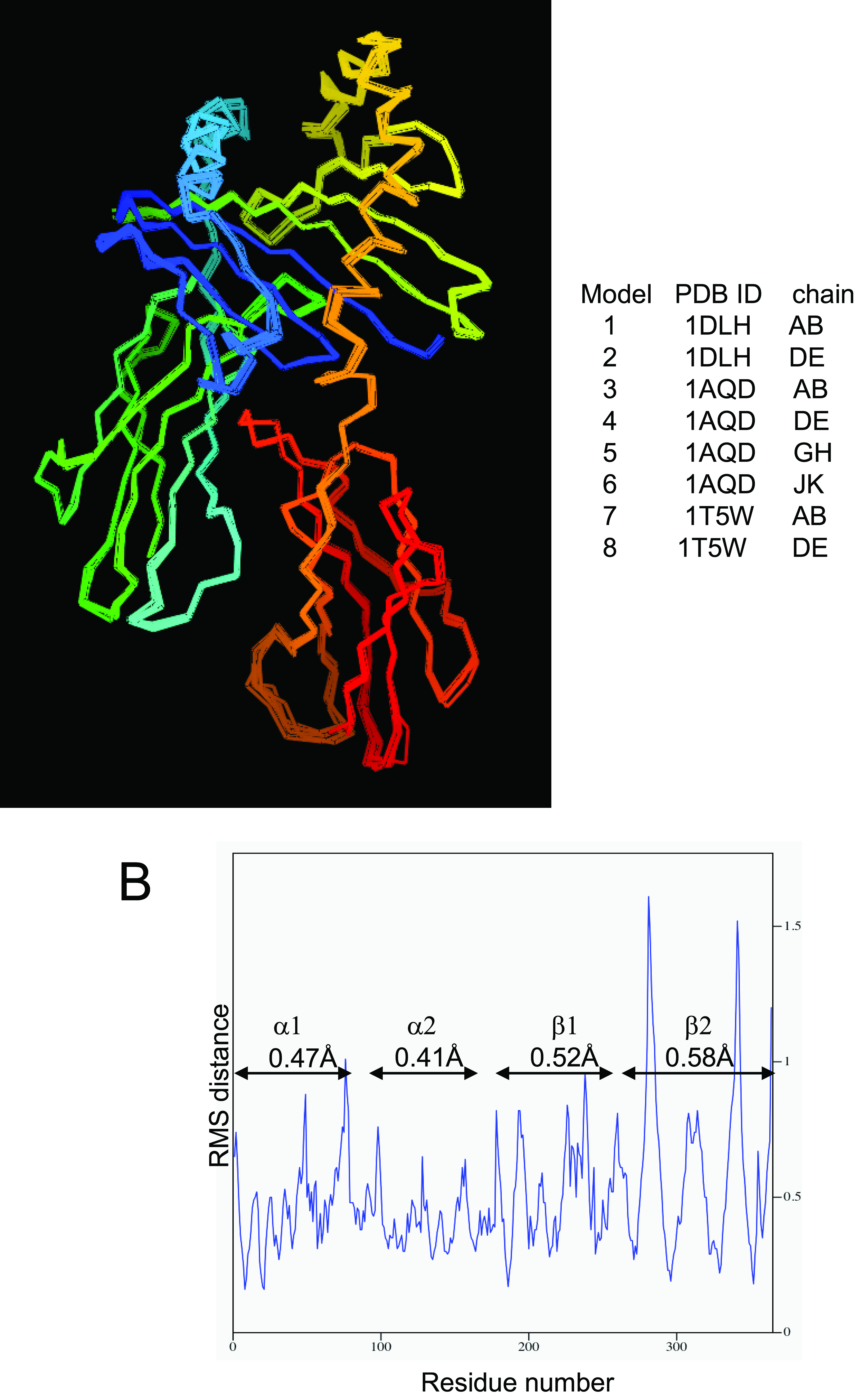

Supplement: Figure S1 — Conformational variation in DR1 crystal structures. A, Overlay of crystallographically distinct αβ heterodimers from three crystal structures reported for DR1-peptide complexes solved in the absence of superantigen. B, RMS Cα distances for aligned αβ heterodimers, with mean RMS in each domain indicated. Structures aligned using LSQMAN [59]. (4.36 MB TIF) [file pone.0002403.s001.tif]

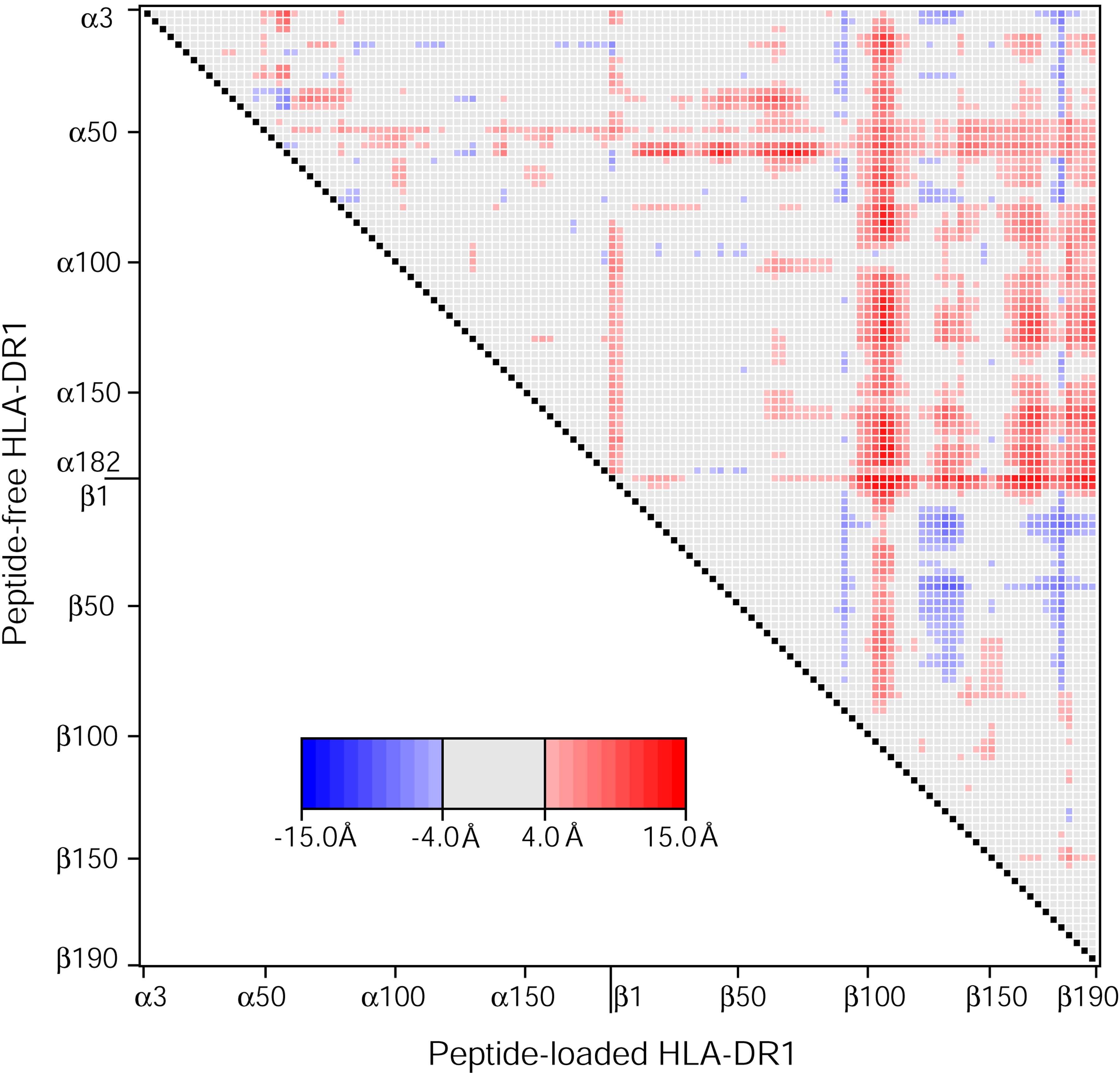

Supplement: Figure S2 — Difference distance matrix between the peptide-loaded and peptide-free conformations of HLA-DR1. The blue squares show areas in the protein that move away from each other in the peptide-free model when compared to the peptide-loaded conformation, with stronger blue intensity for the areas that are the further apart (15 Å difference) and lighter blue for the areas that move away but not as much (4 Å difference). The red squares show regions on the peptide-free form that move closer to other regions in the protein when compared to peptide-loaded HLA-DR1; intense red color are areas that move more. The gray squares are regions that do not change as much (0–3.99 Å). (4.79 MB TIF) [file pone.0002403.s002.tif]

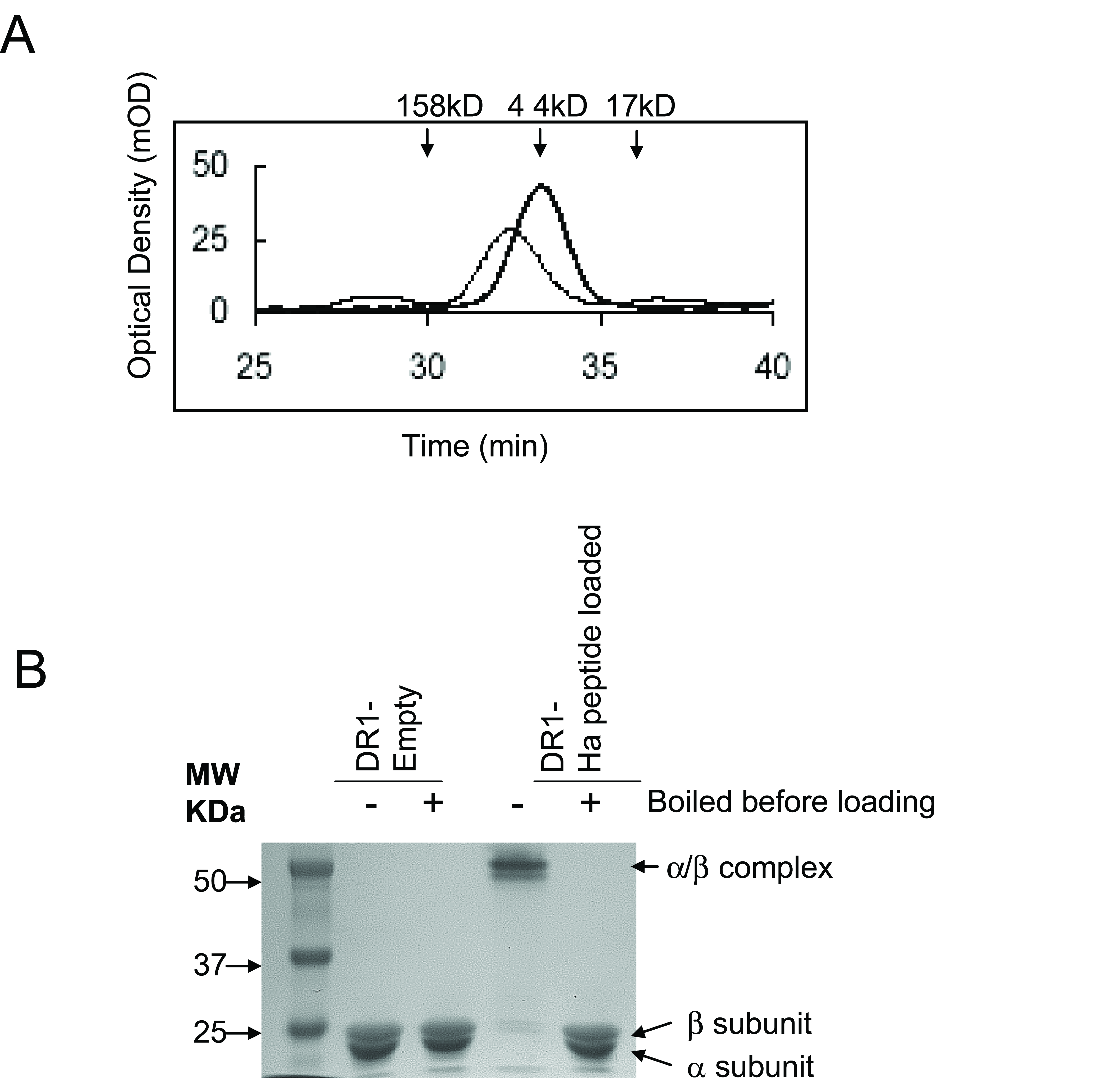

Supplement: Figure S3 — Characterization of peptide loaded and peptide-free DR1. A, analysis of peptide-free DR1 and peptide-loaded DR1 by gel filtration (Superdex 200). Peptide-free DR1 (dotted line) has a larger hydrodynamic radius the peptide-loaded DR1(solid line). Arrows indicate position and molecular weight of standard proteins. X axis represents time in minutes, Y axis represents optical density (milli OD). B, 12% SDS-PAGE analysis of peptide-free DR1 and peptide-loaded DR1. Peptide-free DR1 dissociates into alpha beta subunits in SDS whereas peptide-loaded DR1 is resistant to SDS dissociation until boiled. (3.27 MB TIF) [file pone.0002403.s003.tif]
